# Extracellular Vesicle-Based Drug Delivery Systems for Head and Neck Squamous Cell Carcinoma: A Systematic Review

**DOI:** 10.3390/pharmaceutics15051327

**Published:** 2023-04-24

**Authors:** Karolina Dżaman, Katarzyna Czerwaty

**Affiliations:** Department of Otolaryngology, Centre of Postgraduate Medical Education, Marymoncka 99/103, 01-813 Warsaw, Poland

**Keywords:** drug delivery systems, head and neck squamous cell carcinoma, exosomes, extracellular vesicles, nanoparticles

## Abstract

It is estimated that there are over 890,000 new cases of head and neck squamous cell carcinoma (HNSCC) worldwide each year, accounting for approximately 5% of all cancer cases. Current treatment options for HNSCC often cause significant side effects and functional impairments, thus there is a challenge to discover more acceptable treatment technologies. Extracellular vesicles (EVs) can be utilized for HNSCC treatment in several ways, for example, for drug delivery, immune modulation, as biomarkers for diagnostics, gene therapy, or tumor microenvironment modulation. This systematic review summarizes new knowledge regarding these options. Articles published up to 11 December 2022, were identified by searching the electronic databases PubMed/MEDLINE, Scopus, Web of Science, and Cochrane. Only full-text original research papers written in English were considered eligible for analysis. The quality of studies was assessed using the Office of Health Assessment and Translation (OHAT) Risk of Bias Rating Tool for Human and Animal Studies, modified for the needs of this review. Of 436 identified records, 18 were eligible and included. It is important to note that the use of EVs as a treatment for HNSCC is still in the early stages of research, so we summarized information on challenges such as EV isolation, purification, and standardization of EV-based therapies in HNSCC.

## 1. Introduction

It is estimated that there are over 890,000 new cases of head and neck squamous cell carcinoma (HNSCC) worldwide each year, accounting for approximately 5% of all cancer cases [1]. HNSCC is a complex and challenging cancer to treat, due to its anatomical location. Current treatment options for HNSCC often cause significant side effects and functional impairments, thus the challenge is to discover more acceptable treatment technologies [2,3]. Extracellular vesicles (EVs) carry biological information [4] and can be utilized for HNSCC treatment in several ways, for example for drug delivery, immune modulation, as biomarkers for diagnostics, gene therapy, or tumor microenvironment modulation.

EVs are nanoparticles released by most cell types and play an important role in cell-to-cell communication. According to current research, signals from EVs are transmitted to the target cell through receptor interaction, membrane fusion, and endocytosis/phagocytosis. Based on their origin and size, EVs can be classified into three groups: (a) exosomes (diameter in the range of 30–150 nm), (b) microparticles, micro-vesicles, or ectosomes (50 nm–1 µm), and (c) apoptotic bodies (50 nm–5 µm) [5]. Among the most widely studied EVs are exosomes. EVs are membranous vesicles that are secreted by various cells, such as dendritic cells, macrophages, mesenchymal stem cells, and endothelial and epithelial cells, into the extracellular space. Many studies indicate that EVs can efficiently provide many distinct sorts of cargo to the recipient cell and can be altered to carry specific proteins, lipids and genetic materials, including messenger RNA (mRNA), microRNA (miRNA, miR), and other small non-coding RNAs and genomic DNA (gDNA), from their progenitor cell [6,7,8]. Therefore, EVs are excellent vehicles for the natural targeting and delivery of therapeutics with unique properties. Engineered EVs containing therapeutic agents can modify the activity of cancer cells. These characteristics have led recent studies to analyse EVs, both unmodified and modified or loaded with drugs, for targeted cancer therapy [9].

This review article summarizes the current state of knowledge regarding the possibility of using EVs as drug delivery systems (DDS) in the treatment of HNSCC. We hope that the information obtained, through a comprehensive search of the literature, will contribute to further development and research in this field.

## 2. Materials and Methods

This systematic review follows the Preferred Reporting Items for Systematic Reviews and Meta-Analyses Statement (PRISMA) [10].

### 2.1. Search Strategy and Eligibility Criteria

The literature search for articles regarding membrane vesicle-based DDS for HNSCC was performed using the four following databases: Scopus, Web of Science, MEDLINE (through PubMed), and Cochrane. The search strategies for each database were properly adapted to each database and are presented in Table 1. The last search was conducted on 11 December 2022, in each of the databases. It was not necessary to contact the authors of the retrieved research articles for additional information.

**Figure 1:**
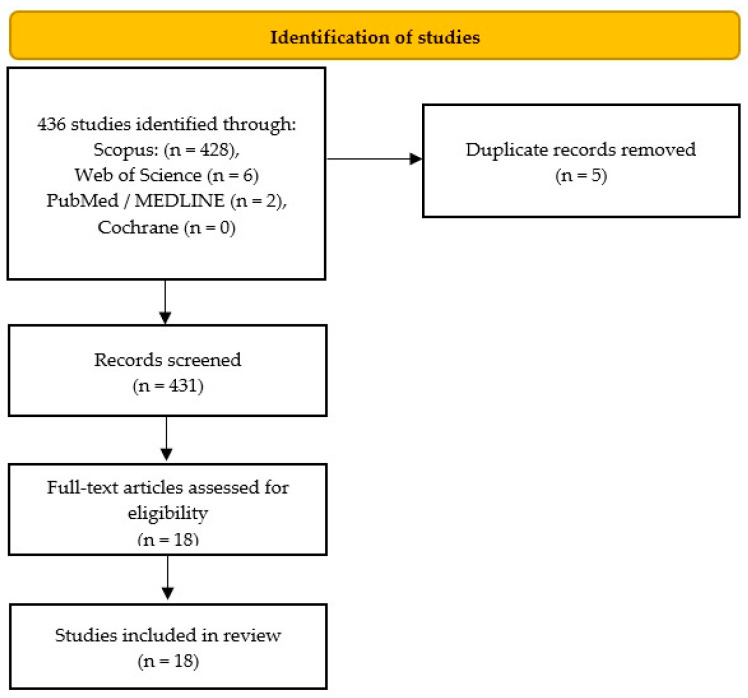
Flow diagram of the systematic literature search.

A systematic literature search identified 436 studies. Duplicates were discarded using the automatic EndNote 10 (Clarivate Analytics, Philadelphia, Pennsylvania, United States) duplicate finder, and then searched manually. Two authors of the study independently screened the records sequentially by titles, abstracts and full-text versions. This systematic review included only English-written, full-text, original research studies regarding membrane vesicle-based DDSs for HNSCC. Figure 1 shows the selection process summarized in a custom-built PRISMA flow chart.

### 2.2. Data Extraction

After evaluation of the eligibility of all included studies, the data for each study were retrieved individually by two reviewers. The following outcomes were taken into account and are presented in this review: baseline information (the first author’s name, year of publication), study design, study and control group, main results, HNSCC model, source of EVs, methods of EVs: purification, isolation, characterization, labelling, tracking, loading, modification for DDS and, finally, therapeutic effects of membrane vesicle-based DDS for HNSCC.

### 2.3. Assessment of Quality of Studies

The risk of bias in data interpretation was assessed using the Office of Health Assessment and Translation (OHAT) [11] and evaluated independently by two investigators. Any discrepancies in judgments regarding the risk of bias were resolved by discussion to reach a consensus between the two review authors. The summarised quality assessment for all studies is reported in Table S1.

## 3. Results

### 3.1. Search Results, Study Characteristics and Study Quality

Details of the selection process are summarized in a custom-built PRISMA flow chart (Figure 1). Upon implementing the search strategy outlined above, a total of 18 articles were identified that met all the inclusion criteria [12,13,14,15,16,17,18,19,20,21,22,23,24,25,26,27,28,29]. The articles were analyzed for their basic data and the main results are described in Table 2, though more detailed information is also presented in Table S2. 

The articles included in this systematic review consist of original studies that were published between 2019–2022, reflecting the recent advancements in this field. Among the selected papers, the majority (13) were conducted in China, while two studies were conducted in Japan, and one each in Israel, France and India. The studies primarily utilized in vivo models, predominantly mice models [12,13,15,17,18,20,21,24,25,27,28,29], in vitro [12,13,14,15,16,17,18,19,20,22,23,24,25,27,28,29], and ex vivo studies [21,26,28].

The risk of bias in the retrieved studies was assessed using the OHAT Tool adopted by the authors for the needs of this review [11]. All included studies had a low to moderate risk of overall bias. The evaluation results are presented in Table S1.

### 3.2. Uniqueness of HNSCC

The vast majority of cancers in the head and neck are HNSCCs originating in the mucosa of the mouth, pharynx and larynx. Unlike other cancers, despite developments in medicine, there is an upward trend in the incidence of these cancers [30].

- Unique risk factors—Cancers for Which a Vaccine Has Been Discovered.

**Figure 2:**
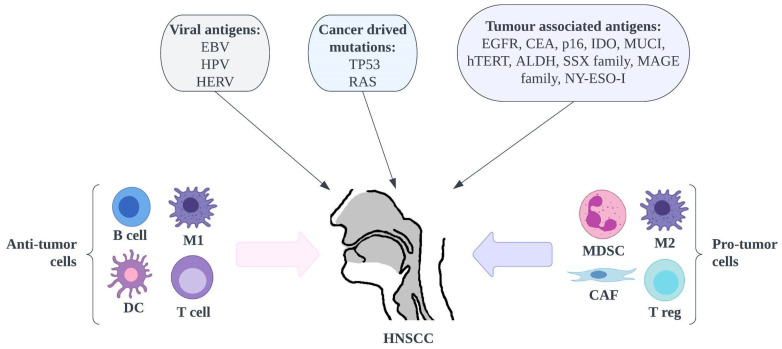
Overview of cancer antigens, immunosuppressive and anti-tumour immune cells in HNSCC. ALDH—aldehyde dehydrogenase, CAF—cancer-associated fibroblast, CEA—carcinoembryonic antigen, DC—dendritic cell, EBV—Epstein-Barr virus, EGFR—epithelial growth factor receptor, HERV—human endogenous retroviruses, HNSCC—head and neck squamous cell carcinoma, HPV—human papillomavirus, hTERT—human telomerase reverse transcriptase, IDO—indoleamine-2,3-dioxygenase, M1—macrophage 1, MAGE—melanoma-associated antigen, MDSC—myeloid-derived suppressor cell, MUCI—mucin-1, NK—natural killer cell, NY-ESO-I—New York esophageal squamous cell carcinoma-1, SSX—synovial sarcoma X, T reg—T regulatory cell.

Main risk factors for these cancers include smoking, alcohol abuse, and oncogenic viruses such as Epstein-Barr virus (EBV) and human papillomavirus (HPV), whose role in this disease has been gaining importance in recent years [31,32] (Figure 2). Because HPV is mainly transmitted to the upper airway through oral sex, the unique feature of HNSCC is an association with sexual behavior. The most oncogenic types are HPV-16 and HPV-18. The importance of HPV is increasing, especially in populations where HPV vaccination has not been introduced [33]. To date, the FDA has approved three prophylactic vaccines against HPV—bivalent, quadrivalent and non-valent, designed for specific age groups and genders. Unfortunately, they are only effective for primary prevention.

Genetic factors are also important in the development of HNSCC. A highly increased risk of developing HNSCC is observed in patients with Fanconi anemia (associated with mutations in the FANC genes) [34]. Additionally, polymorphisms of other genes, e.g., in CTLA4 [35] or CYP1A1 [36], cause an increased incidence of these cancers.

It has also been observed that there is an increased incidence of these cancers in certain geographic areas, which is associated with cultural factors very specific to HNSCC, such as the chewing of areca nut products in the Asia-Pacific region [1]. Another important risk factor for HNSCC is exposure to air pollution, which is particularly intense in Asia [37].

B. Specific Biomarkers for HNSCC and Second Primary Tumors.

To date, many biomarkers with prognostic significance in HNSCC have been discovered. Among the most important are CD44, CD133 and ALDH1, high levels of which are associated with metastasis, invasiveness and poor prognosis [38,39].

A huge problem concerning patients with HNSCC is that the diagnosis of this disease is most often made at a late stage, with a high local stage of the disease and the presence of metastases to regional lymph nodes [40]. Moreover, a very distinctive feature of HNSCC is an association with the development of second (or even third or fourth) primary tumors, which are most often located in the head and neck, lung and esophageal regions [41], that has to be considered during diagnosis.

C. High Suicide Rate and Poor Quality of Life.

However, the most important distinguishing factor of HNSCCs is their critical location. The head and neck region is responsible for the most important life functions such as eating, breathing, communication and social relations. There, all sense organs responsible for receiving stimuli from the outside world are situated. So, HNSCC diagnosis and treatment are most likely to be related to the significantly reduced quality of life. Therefore, unfortunately, patients with HNSCC, after pancreatic cancer patients, have the highest suicide rate (63.4 suicides per 100,000 patients) among oncology patients [42].

When treating patients with HNSCC, special attention should be paid to the possibility of preserving the function of affected organs. Patients after surviving this disease complain of a significantly reduced quality of life and disabilities such as swallowing, speech and voice problems [43,44,45].

### 3.3. Head and Neck Squamous Cell Carcinoma Model

Several authors have demonstrated EVs potential for therapeutic drug delivery in HNSCC. The majority of these studies focussed on oral squamous cell carcinoma (OSSC) (Table S2) and were based on both in vitro and in vivo models. In three research papers, only in vitro analysis was performed, using cell lines transfected with specific EVs [14,19,23].

In most publications, the xenograft OSSC mice model was used to design the in vivo study, and in one article the hind model [27].

Interestingly, some investigators also performed ex vivo studies and developed an ex vivo tumour spheroid model to check the efficacy of the proposed therapeutic approach [21,26,28]. The 3D tumour spheroid models have changed the typical processes of cancer research. The engineered 3D human tumour models using carcinoma cells in polymeric scaffolds recreate microenvironmental characteristics representative of tumours in vivo [5]. They recapitulate several aspects of the tumour microenvironment (TME), especially critical stroma components, and provide better predictive results than 2D culture monolayers used in in vitro studies. Thus some researchers treat 3D ex vivo models as in vivo models. Using a 3D model, the authors demonstrated that the presence of stroma content in 3D HNSCC spheroids strongly affected the uptake of NPs (nanoparticles), while penetration and photodynamic therapy (PDT) efficacy were less sensitive to the presence of stromal components. To sum up, the 3D tumour models mimic heterogeneity and tumour pathophysiology, bridging the gap between 2D cultures and in vivo testing [9].

### 3.4. Carriers for Head and Neck Squamous Cell Carcinoma Drug Delivery Systems

The use of nanotechnology and, more specifically, drug delivery is a growing field of medicine and biotechnology. Despite convenience for the patient, traditional oral administration of anticancer drugs is often impossible due to their poor water solubility, so they are delivered intravenously. Most anticancer agents can also be administered via other means of delivery when incorporated in suitable carrier (bio)materials. Carrier-based DDSs allow for the controlled release of drugs, enhance selectivity and effectiveness, and reduce the side effects of chemotherapy. Currently, there are DDSs based on NPs, nano lipids, hydrogels and, recently, widely investigated EVs. Here, we focus on membrane vesicle-based DDS for HNSCC and the application of different types of cell-derived EVs for cancer therapy.

### 3.5. Membrane Vesicles for Drug Delivery Systems

#### 3.5.1. Source of Extracellular Vesicles

Membrane vesicles for DDS can come from various types or parts of cells, including EVs. The membrane vesicles for DDS encompass a variety of types, such as apoptotic bodies, exosomes, microparticles, outer membrane vesicles, or cell-bound membrane vesicles isolated from cell surfaces.

EVs have been identified from several sources [12,28,29,46,47]. However, it is important to consider that EVs derived from different sources have diverse applications in EV-based cancer treatment, because they reflect the functional status of their parent cells. The EVs’ parent cell origin determines membrane composition and bioactive cargo.

##### γδT Cell-Derived Extracellular Vesicles (γδTDEVs)

It is reasonable to assume that EVs derived from γδT cells, dendritic cells (DCs) and natural killer (NK) cells could inherit the direct cytotoxicity and antigen-presenting role and be considered for cancer therapy [46,47,48]. The γδT cells represent a minor lymphocyte population (about 0.5–16% of total CD3+ cells) and, when isolated from patients with cancers, efficiently killed tumour cell lines, played a role at antigen-presenting cells (APCs) and induced CD4+ and CD8+ T cell proliferation and cytotoxicity. Two included studies [17,25] reported γδTcell-derived EVs’ (γδTDEVs’) advantages mentioned above, such as direct cytotoxicity against cancer cells and antigen-presenting function, but also adopted a large-scale expansion protocol for γδ T cell production [49]. The authors confirmed that the specific features of γδ-T-EVs may potentially play a role in nasopharyngeal carcinoma (NPC) therapy [25]. As demonstrated previously, NPC cells secrete abundant CCR5 chemokine ligands and TGFβ in order to suppress T-cell responses [50]. The authors revealed that γδ-T-EVs promoted T-cell migration into NPC by upregulating CCR5 on T cells. In consequence, γδ T cells were chemoattracted by CCR5 ligands in TME. Moreover, γδ-T-EVs preserved T-cells’ direct antitumor activity in NPC and effectively killed both NPC EBV positive and negative cells and reduced the tumour size by around 80%. This reaction was mediated by Fas ligand (FasL) and DR5/TRAIL pathways (DR 5—death receptor 5, TRAIL—tumour necrosis factor-related apoptosis-inducing ligand).

Additionally, they demonstrated that γδ-T-EVs induced significant cell apoptosis and selectively targeted radioresistant CD44+/high cancer stem-like cells (CSCs) [50]. Thus, combined therapy using irradiation and γδ-T- EVs increased NPC cell apoptosis when compared with monotherapies.

In another study, γδ-T-EVs used in OSSC therapy [17] were additionally loaded with miR-138 and had dual anti-tumoral functions. They immune-stimulated both CD3+T and CD8+ T cells and impacted cell cycle and proliferation by gene regulation. Furthermore, the researchers revealed that miR-138 γδ Tcell-derived EVs were more effective than miR-138-transfected liposomes.

Taken together, these data indicate that γδ-T-EVs can promote T-cell migration into the TME of HNSCCs, preserve their tumour-killing activities, and overcome their radio-resistance.

##### Macrophage-Derived Extracellular Vesicles (M-EVs)

Recent studies have shown that macrophages are fundamental for promoting tumour genesis, development, metastasis and chemoresistance through modulating TEM and cancer cells. Macrophages are the most infiltrative immune-related stromal cells [51] and can be polarized into classical M1 or M2. Some authors noticed that HPV + HNSCC-EVs were able to transform macrophages into the M1 phenotype, which subsequently increased the radiosensitivity of HNSCC [22].

Moreover, M-EVs show heterogeneity in various cancers and play paradoxical roles in tumour-suppression and promotion, mainly through post-transcriptional control and regulation of protein phosphorylation in the recipient cells [52]. Li [16] used M-EVs as a biomimetic strategy to coat and protect the miR-144/451a cluster from the immunological system, and improved its delivery to OSCC cells.

##### Mesenchymal Stem Cell-Derived Extracellular Vesicles

The most common sources of EVs are mesenchymal stem cells (MSCs), due to their multipotential in differentiating into several cell types. MSC-EVs are involved in intercellular communication through the transfer of proteins, RNA, DNA and bioactive lipids, and thus are implicated as the mediator of many therapeutic potencies. Both natural or engineered EVs derived from MSCs or tumour cells with different origins have been previously investigated in cancers such as pancreatic cancer or breast cancer and used as DDS [53,54]. MSCs have been successfully isolated from numerous tissues, including bone marrow, adipose, peripheral blood, skin, brain, dental tissues and endometrium. In this systematic review, some authors explored the therapeutic potential of MSC-EVs derived from human umbilical cord MSCs [12,26,28], but also the subtypes of immature MSC—SHED cells (stem cells of deciduous exfoliated teeth) [18].

Compared to other EV sources, MSCs are characterized by higher EV production, good stability in vivo, high targeting specificity, strong ability to proliferate and easy accessibility as a source of stem cells [55].

Moreover, MSCs have been genetically engineered using viral and nonviral vectors to improve host immune response against cancer cells and to carry traditional anticancer cytotoxic drugs. The viral vector-lentivirus expressing human TRIAL was used in two included studies to engineering transfected MSC (MSCT) [18,20].

##### Carcinoma Cell and Normal Cell Lines—Derived Extracellular Vesicles

Recently, several studies have demonstrated that tumour-derived EVs (TEVs) have potential in immunomodulation and play an important role in the escape of tumour cells from immune surveillance. It has been observed that TEVs can activate CD4+ T cells to promote the mitochondrial apoptotic pathway [56].

In revised in vitro studies, the investigators used EVs derived from several carcinoma cells, such as the human epidermal carcinoma cell line (A431-exo) [12], the oral adeno-squamous carcinoma cell line (CAL 27) [27], the human cisplatin-resistant and cisplatin- sensitive OSCC cell lines (UPCI-SCC-131) [21], and the HPV positive HNSCC (HPV + HNSCC-EVs) [22].

Other authors have explored EVs derived from normal tongue epithelial cells (NTECs –exo) [13], human embryonic kidney cells (HEK293T cells) [14,23], or octEVs—engineered OSCC-targeted EVs which were constructed based on human skin-derived fibroblasts transfected with EBV [48].

##### Plant-Derived Extracellular Vesicles

EVs are present in biological fluids from mammals, such as blood, urine and plasma. However, many disadvantages slow down the translation of autologous EVs, such as limited yields, longer drug preparation time, cancer-stimulating risk and ethical problems. Therefore, techniques for obtaining EVs from other sources are being developed [28,29].

For example, it has been found that plant–derived EVs play a role in regulating intestinal homeostasis and cell-to-cell communication, but also may exhibit anti-inflammatory and anti-cancerous effects [57]. Furthermore, some data suggest that other tissues in the body may be affected by dietary PDEVs. It has been noticed that a clear preference for specific organs depends on the source of the PDEV [58]. While plant-derived EVs have been shown to have numerous benefits, they also have some limitations. The main challenges are the protocol standardization and characterization of PDEVs [59]. Only one reviewed study explored the potential of plant-derived EVs for HNSCC treatment [28]. The authors revealed that bitter melon-derived EVs (BMEVs) suppressed OSCC proliferation, induced S phase arrest, and persuaded apoptosis via ROS-mediated mitochondrial injury (ROS—reactive oxygen species). BMEVs significantly downregulated NLRP3 (NOD-like receptor family pyrin domain containing 3) expression. The NLRP3 activation contributed to the resistance of OSCC cells to 5-FU. Therefore, BMEVs demonstrated the synergistic therapeutic effects of 5-FU against OSCC, both in vitro and in vivo. Twenty-four kinds of miRs were detected in BMEVs and 11 had the potential to regulate the expression of NLRP3 mRNA.

##### Bovine Milk-Derived Extracellular Vesicles

Bovine milk-derived EVs were firstly isolated in 2010 [60]. These EVs have been proven to reduce the systemic toxicity of free chemotherapeutics and improve antitumor effects against lung and breast cancers. Moreover, they have been orally administered, because bovine milk-derived EVs can pass through the gastrointestinal barrier. Interestingly, despite the reduction in primary tumour growth, milk-derived EVs accelerate metastasis in breast and pancreatic cancer mouse models. Surprisingly, the timing of EV administration after resection of the primary tumour was critical in reversing their pro-metastatic effects [61]. Bovine milk-derived EVs have the advantages of both synthetic and cell-mediated nanocarriers. Zhang [29] used milk-EVs to develop a new sensitive DDS loaded with doxorubicin (Dox) for photochemistry therapy against OSCC. Milk-EV technology has been proven to reduce normal tissue toxicity from Dox.

To sum up, it needs to be highlighted that the EVs’ source impacts their targeting abilities, EV migration, biodistribution and tumour accumulation. Thus, before EV engineering, it is crucial to choose the appropriate EV source as a starting point. The most common sources of EVs in the research field are MSCs, due to their multipotent nature and their ability to differentiate into various cell types. From this point of view, they are excellent candidates for cell-based therapy. On the other hand, their expression in tumour cells and promotion of tumour growth in vivo could be controversial in their usage as EV source. Therefore, some researchers indicate that the best sources of EV are cytotoxic cells against cancer cells, such as γδ T cells or macrophages. Recently, more attention has been paid to new EV sources, such as plants.

#### 3.5.2. Extracellular Vesicles—Purification and Isolation

One of the basic conditions for the clinical use of EVs is the standardization of the isolation process, increasing its efficiency, repeatability and purity. In the case of large-scale EV production, the production process sequentially involves the expansion of the donor cell line, collection of the conditioned medium and EV purification [62]. To date, several approaches to EV isolation have been reported: differential centrifugation (ultracentrifugation, UCG), precipitation, flushing separation, ultrafiltration, antibody affinity capture, microfluidic separation and mass spectrometry. The UCG is currently the most available method, considered the “gold standard” for EV isolation, and was used in almost all studies included in the review (Table S2). While UCG is the most popular and cost-effective procedure, there is a risk of disrupting EVs beyond a certain speed and force, which must be taken into account [63]. The latest evidence has shown that, compared to other methods, three-time UCG is a time-consuming procedure, may result in a lower yield of vesicles, and raises the problem of co-contamination with unwanted proteins [64]. Therefore, a double UCG scheme has been proposed that involves the addition of 30% sucrose buffer solution during the first centrifugation step [65]. Summarizing, the final result of UCG may be affected by centrifugation speed, force, and type of rotor.

Only in one study [18] was the precipitation method applied, based on the EV isolation kit and addressing EVs. EV purification kits enable the isolation of EVs from all major biofluids, including plasma, and are considered as simple methods for EV isolation This method delivers comparable results to UCG without the necessity for specialized equipment.

#### 3.5.3. Extracellular Vesicles’ Characterization

As mentioned above, EVs can be divided into three types, depending on their size. To clearly understand the role of EVs, it is essential to assess their physical and chemical properties, such as shape, size, surface charge and density. Characterization of EVs to establish their role in physiological and/or pathological processes is one of the major challenges in the EV field. Many technologies are frequently applied to characterize EVs, among them dynamic light scattering (DLS), transmission electron microscopy (TEM), scanning electron microscopy (SEM), atomic force microscopy (AFM), tuneable resistance pulse sensing (TRPS), nanoparticle tracking analysis (NTA), flow cytometry (FCM), and confocal microscopy (LSM—confocal laser scanning) [66]. Most of those techniques were used in the reviewed studies (Table S2).

In the majority of articles, the size and structural morphology of EVs were confirmed by TEM and showed a typical bilayer membrane. Exosomes, which have a uniform round cup shape and a diameter of 30–150 nm, can also be observed by SEM. These features distinguish them from apoptotic bodies and micro-vesicles. The major distinction between SEM and TEM technology is that SEM generates an image by detecting reflected or knocked-off electrons, whereas TEM uses transmitted electrons (electrons that are passing through the sample) to create an image. As a consequence, TEM provides precious details regarding the internal structure of the sample, such as crystal structure, morphology and stress state information, while SEM delivers information on the surface of the sample and its composition [67]. Few researchers have assessed the particle size of EVs using DLS [24,29] or LSM [24,27].

In addition, in all studies, the western blotting analysis confirmed the existence of typical membrane proteins of the EVs, including well-known markers. In mammalian cells, CD63, CD81 and CD9 are among the most common positive markers, and they were the most frequently used in the reviewed studies, and also TSG101, while negative exo-somal markers were, unfortunately, reported only in four papers pointing to calnexin [14,17,22] and GM130 [13].

It has to be mentioned that, depending on the origin cells, the markers for detection may vary, therefore for PDEV identification authors [28] used other specific markers, such as heat shock protein 70 (HSP70), S-adenosyl-homocysteinase and glyceraldehyde 3 phosphate dehydrogenase.

#### 3.5.4. Extracellular Vesicles’ Labeling and Tracking

EV labelling is a novel strategy for monitoring EV distribution in vivo, which contributes to optimizing EV-based diagnosis and treatment. EV tracking helps in determining their applicability as site-specific delivery vesicles and their half-life, biodistribution and migration abilities, but also to understanding their role in intercellular communication [68]. Recent studies have developed different methods for EV labelling and imaging. Simple and widely used tracking is performed by lipophilic labelling of EVs, either using fluorescent dyes or radiolabelled dyes [69,70].

Direct fluorescence tagging of EVs is extensively used to study the in vivo behaviour of exogenous EVs. Fluorescence labelling can provide whole-body images in highly sensitive optical cameras, as well as fluorescence microscopic images [71]. Fluorescence dyes including green fluorescent protein (GFP), or lipophilic substances like PKH, DiI, and DiR are simply and commonly used to track EVs [72], and this is the most commonly applied strategy in the reviewed studies (Table S2). The marking procedure is quite simple and no genetically modified EVs are needed. Nevertheless, optical imaging is restricted to exogenous EVs, and fluorescent dyes remain in tissues even after the degradation of EVs [73].

Another method is labelling used protein markers of EVs, such as CD63, which could be conjugated into fluorescent proteins. Reporter imaging using fluorescence or bioluminescence combined with transmembrane proteins could provide information more specific to EVs than direct dye labelling [53].

For clinical applications and deep tissue imaging, radionuclide imaging or MRI are possible, using 111In-oxine, 99mTc-HMPAO and iron oxide NPs.

Some researchers used gold NPs (GNPs) as labelling agents, creating golden EVs with the superior visualization abilities of classical X-ray computed tomography (CT) [13,21,74]. These authors developed a technique for direct and highly efficient uploading of GNPs into EVs, based on glucose-mediated uptake, without affecting EVs’ functionality. Subsequently, the golden EVs were tracked with CT and quantitatively analyzed, in order to reveal their tumour targeting, accumulation, and whole-body biodistribution.

In some cases, chemotherapeutic drugs loaded in EVs, such as Dox, are intrinsically fluorescent and used in distribution assessment [29]. The uptake of free Dox or NPs loaded with the drug can be observed at 594 nm using LSM after excitation at 480 nm.

However, accurate tracking of EVs is limited due to the non-specificity of the labelling and retention, or the recirculation of labels after degradation. From the practical point of view, CT or MRI imaging could be more useful for EV tracking compared to fluorescence imaging, because of better resolution and penetration abilities and cost-effectiveness [68,71]. Compared to nuclear imaging, a CT scan allows tracking of EVs for longer periods than MRI. Using CT, but also immunohistochemical staining for human CD63, Cohen [12] tracked golden EVs at 24 h and 48 h after injection to the HNSCC model. Thanks to EV labelling, the authors confirmed that MSC-EVs had higher tumour accumulation compared to A431-EVs, and to synthetic NPs.

Some scientists have also used an in vivo imaging system that combines 2D optical and 3D optical tomography in one platform, known as IVIS imaging [15]. Moreover, tracking EVs can be provided using a different type of microscope, such as LSM [15], SME, or a light microscope, where the accumulation of gold-loaded EVs is observed during histological analysis by dark field microscopy [12].

#### 3.5.5. Modified Extracellular Vesicles for Drug Delivery

Various nano-based drug formulations have been used to improve the therapeutic efficacy of chemical and biomolecular drugs. EVs are easy to be surface modified, which delivers additional functionality to the EVs and is an emerging area in drug delivery research.

##### Surface-Modified Extracellular Vesicles

On the surface of the EVs’ membrane are localized particles which play role in EV protection, tumour targeting, cell adhesion, and uptake, which can be easy to change. Some authors have examined the modification of MSCs to express membrane-binding TRAIL [18,20]. They used MSCs transduced with a lentiviral vector encoding TRAIL to construct MSCT (transfected MSC) which subsequently delivered EVs expressing TRAIL. This nanotechnology can enhance the tumour-targeting effect of TRAIL [20]. Afterwards, the authors prepared a combination of MSCT-EVs (containing TRAIL) with drugs: CTX [20] or SNS032 [18]. The investigators found that the MSCT-EVs system improved the apoptosis index in tumour cells, showed a high inhibitory effect on OSCC, reduced the dosage of free drugs and decreased the side effects of cancer therapy.

Others [15] have transfected EVs with an EBV vector using a Neon electroporator to express a transmembrane protein (EBV Inducted 3 (EBI3) on their membranes, which is abundantly expressed by OSCC cells. As a result, they improved EVs’ effectiveness in inhibiting tumour cells. Similarly, the EBV vector was used in NPC to express avb3 integrin subunits on the NPC surface [25]. This integrin binds iRGD, so subsequently EVs were modified to express iRGD, which significantly improved the binding ability of EVs to NPC and the biodistribution of iRGD-EVs.

To sum up, the targeting ability of EVs can be upgraded through appropriate surface modification leading to greater accumulation in tumour cells.

##### Extracellular Vesicles Combined with pH-Sensitive Peptide for Drug Delivery

It is known that the majority of solid tumours exhibit a pH from 6.5 to 7.4, thus giving the possibility of creating pH-response NPs [75]. The EV membrane can be conjugated with chemotherapy drugs with a hydrazine bond, which is easily disintegrated in an acidic TME, resulting in the quick release of drugs at the tumour site. This method was used by [29] to engineer pH/light sensitive milk-EVs, conjugated to Dox by a pH-cleavable bond. Afterward, the milk-EVs/Dox was used to encapsulate two agents: anthracene endoperoxide derivative (EPT1) and chlorin e6 (Ce6), finally creating EVs@Dox–EPT1 (EV–doxorubicin–anthracene endoperoxide derivative NPs). When the NPs were at the tumour site, Ce6 generated plasmonic heat and accelerated singlet oxygen generation from EPT1 under NIR irradiation. As a result, reliable and tumour site-specific photochemistry was realized in the treatment of OSCC [29].

EV engineering can improve phototherapy techniques and address their limitations. The latest research has focused on incorporating chemotherapeutic agents and photosensitizers or light-absorbing agents into NPs. When these agents are delivered to the tumour site, local irradiation has been shown to kill cancer cells and shrink the tumour.

##### Extracellular Vesicle Capturing Used in Biomimetic Strategy

The biomimetic strategy is a new technology that extracts the inflammatory cell membrane and uses it to coat nucleic acid or NPs in order to avoid the monitoring of the immune system which prolongs circulation time [76].

This strategy was used by Li [16], who camouflaged chitosan NPs (CAs) with macrophage membranes derived from M-EVs. Chitosan is an important non-viral gene vector that forms a complex with anionic drugs to improve delivery. Firstly, they constructed CAs using the ionic cross-linking method, along with biomimetic nanoparticles coloaded with the miR-144/451a cluster. Next, M-EV coated CAs and, finally, (M-EVs)/CA-miR451a construction were successfully applied for OSCC treatment.

##### Engineering Extracellular Vesicles by Fusion with Liposome

Another innovation is EV fusion with liposomes, which improved the loading capacity and the targeting capability of EVs. Liposomes are structures that consist of single or multiple concentric lipid bilayers encapsulating an aqueous compartment. They are able to incorporate drugs both within their aqueous core and their lipid bilayers. Fusing the EV membranes with liposomes using the freeze–thaw method creates the possibility of transporting exogenous hydrophobic lipids and hydrophilic cargoes.

##### Extracellular Vesicle-Coated Metal-Organic Framework Nanoparticles

Metal-organic frameworks (MOFs) are a type of organic–inorganic hybrid compound with one-, two-, or three-dimensional (1D, 2D, 3D) structural topologies, made up of inorganic metal ions/clusters and organic ligands [77]. The result shows that EV-coated MOF NPs are a smart and efficient DDS with an “onboard trigger”.

##### ExomiR-Tracker—A Novel Drug Delivery System

In the reviewed studies, a novel DDS using ExomiR-Tracker [27] was described. ExomiR-Tracker consists of anti-exosomal miR-21 oligonucleotides and anti-exosomal CD63 antibodies creating an anti-CD63-9r/anti-miR complex. Anti-miR oligonucleotides have a sequence complementary to miRs and are often used for their functional inhibition [78]. Anti-miR-21 exhibits specific inhibitory effects on gene expression and, under hypoxic conditions, is upregulated, promoting cell growth and migration [79]. ExomiR-Tracker binds onto the surface of the EVs, leading to incorporation into the cells and the subsequent inhibition of the function of the exosomal miRs in the recipient cells. This strategy allows the use of EVs as natural cargo without isolation and loading [80].

##### Comparison of Methods for Drug Delivery Systems

DDS preparation is based on chemical methods (viral and non-viral vectors or liposomes) and physical techniques (such as nuclear transfection and electroporation). However, all these methods may cause significant damage to cells, thus their application in vivo is limited [18]. Common viral vectors, such as adenovirus (AV) and retrovirus (RV), are characterized by high-efficiency gene transfection, but there are immunogenicity and biological safety problems. Non-viral vector has become a breakthrough in the research of gene-drug delivery due to advantages such as simple preparation, low toxicity and immunogenicity, but also unlimited size and type of genes [51]. Additionally, non-viral vectors allow the co-delivery of different drug, or the combination of gene therapy with chemotherapy.

Some studies assessed the difference in drug (mTHPC) penetration and efficiency between three types of DDS: conventional liposomes (Foslip^®^), drug-in-cyclodextrin-in-liposomes (DCL), and EVs [26]. In the Foslip^®^ and DCL methods, the mTHPC is directly loaded in lipid suspension. In contrast, mTHPC-EVs’ construction requires the incubation of HUVEC cells in the serum-containing free mTHPC. However, mTHPC aggregates stay inside EVs even after purification and isolation procedures. The assessment of mTHPC loading demonstrated the highest loading capacity in EVs compared to DCL and Foslip^®^ (1.3 × 10^−15^ g, 0.07 × 10^−15^ g, 0.05 × 10^−15^ g, mTHPC per nanovesicle, respectively) which resulted in mTHPC-EVs outperforming other lipid nanovesicles. The total uptake of mTHPC-EVs in the PSCC model was 10 times higher than that for other NPs, so tumours exposed to mTHPC-EVs required a four-times lower light dose to obtain a similar therapeutic effect in photodynamic therapy (PDT) as mTHPC-DCL and Foslip^®^.

To sum up, investigations confirmed that EVs most effectively improve drug delivery to cancer cells due to their extremely high loading capacity in comparison to other lipid nano-vesicles and allow for a decrease in therapeutic dosage.

#### 3.5.6. Loading of Extracellular Vesicles

The lipid bilayer of EVs protects cargo from degradation in the bloodstream but, unfortunately, makes EV loading challenging.

Several strategies have been proposed and developed for loading material into EVs, e.g., electroporation, sonication, freeze–thaw cycles, and a combination of incubation with permeabilizing agents which have been lipophilically modified (Table S2). Each of these methods has some drawbacks. The disadvantage of electroporation is precipitation and aggregation, leading to an overestimated loading efficiency for EVs [69].

During sonication and incubation, the reorganization and deformation of EVs may occur, which disturbs their integrity [70]. Some researchers consider that charging hydrophobically modified cargo onto EVs is an optimized method. This was used by Deng [14], where cholesterol-modified miR-34a was loaded into EVs of HEK293T cells by co-incubation, without impacting EVs’ integrity. The authors found that incubation provides a robust, efficient and highly reproducible method for loading EVs [14,26].

Some authors have used lentiviral vectors [13,17,19,20] or EBV vectors [15]. After transfection of the mother cells, such as MSCs, T cells, fibroblasts, or other epithelial cells, using the miR-encoding vector, it was possible to isolate EVs secreted from the transfected mother cell with overexpression of miR.

A modified calcium chloride-mediated transfection method for loading the miR inhibitor has also been performed [21,81]. This method is used to load miR directly into isolated EVs, instead of transfecting their mother cells.

### 3.6. Therapeutic Effects of Membrane Vesicle-Based Drug Delivery System for Head and Neck Squamous Cell Carcinoma

**Figure 3:**
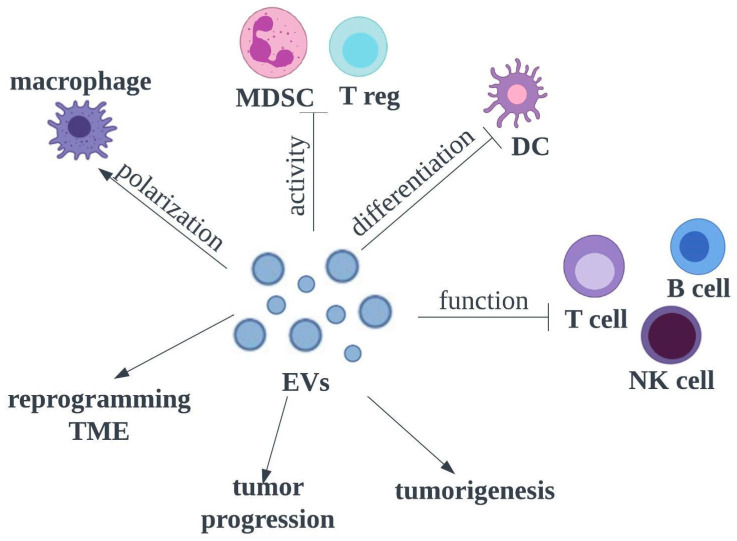
Role of EVs in the tumour microenvironment and impact on the immune system in HNSCC; DC—dendritic cell, MDSC—myeloid-derived suppressor cell, NK—natural killer cell, T reg—T regulatory cell, TME—tumor microenvironment.

EVs have a cross-curricular role in tumor-genesis, cancer progression and immune response by transferring bioactive molecules between the cancer and various cells in the local and distant microenvironments, but are also being used as DDS (Figure 3).

#### 3.6.1. Evaluation of Treatment Effectiveness

Cell viability, migration, invasion and wound-healing assay were performed to evaluate DDS efficacy, based on commonly used methods. Cell viability was assessed using the CCK8 Assay Kit. Cell migration and invasion abilities were determined using the Transwell migration assay. To evaluate cell motility, scratch wound healing assays were accomplished and immunofluorescence staining of Ki67 was performed to examine cell proliferation ability. Furthermore, researchers have investigated whether EVs induce apoptosis, which is recognized as a crucial type of cell death, using flow cytometric analysis via annexin V-FITC/PI staining. Chemotherapy effectiveness was revealed by increasing the percentage of apoptotic cells. Moreover, western blotting was performed to confirm the involvement of caspase activity in the EV induced apoptosis, because the activation of caspase pathways is usually involved in apoptosis induction.

#### 3.6.2. Anticancer Agents Derived from Extracellular Vesicles for Head and Neck Squamous Cell Carcinoma Treatment

The following are the first-line chemotherapy drugs for treating HNSCC: paclitaxel (PTX), cisplatin (DDP), doxorubicin (Dox), docetaxel (DTX), Methotrexate and Fluoropyrimidine 5-Fluorouracil (5-FU). Four chemotherapeutics from this group were investigated in the reviewed studies: Dox [29], DTX [13], DDP [21] and 5-FU [28]. Additionally, in one article, Cabazitaxel (CTX), which is mainly used in prostate cancer, was under consideration [20].

The systemic toxicity of commonly used chemotherapeutics to treat HNSCC is a major complication of clinical treatment. It is known that the molecular mechanisms of chemoresistance include the acquisition of stem-cell-like features, epithelial-to-mesenchymal transition (EMT), overexpression of multidrug resistance (MDR) gene-related ATP-binding cassette (ABC) transporters, and activation of DNA repair mechanisms [81]. The responsiveness of cell carcinoma to chemotherapy widely affects prognosis. Moreover, the chemoresistance of cancer cells requires higher drug doses, which increases side effects. Therefore, ways to reduce doses in anti-tumour therapy are being investigated. This can be achieved by combining a chemotherapeutic agent with a chemosensitizer, or by DDSs directly to cancer cells. These obstacles can be overcome by using DDS targeting cancer cells that improve drug accumulation in tumours.

The most common drugs family loaded in EVs are taxane anticancer drugs, such as CTX, PTX and DTX, which bind to tubulin and subsequently suppress microtubule dynamics in cell division, leading to cancer cell death. Moreover, taxol drugs inhibit the apoptosis of cancer cells and promote cell proliferation, inhibiting the phosphorylation of PI3K, Akt and mTOR [63,82,83,84,85]. A therapy based on MSCT-EVs loaded with CTX/TRAIL combination was assessed for OSCC treatment [20]. CTX demonstrates antitumor activity superior to that of DTH and is mainly used to treat tumours with resistance to PTX or DTX, due to its poor affinity for P-glycoprotein (P-gp). It adverse effects, poor stability, and poor aqueous solubility impede its clinical applications. The authors compared the anti-tumour effectiveness of CTX-free, free MSCT-EVs and CTX-loaded by MSCT-EVs and observed that MSCT-EVs/CTX were the most effective in killing cancer cells. Finally, they concluded that EVs not only prolong the blood circulation time of CTX and improve its biological activity, but also decrease its toxicity in patients.

The same MSCT-EVs engineered with TRAIL were also used to load SNS032 [18]. SNS032 is known as a new and effective selective CDK9 inhibitor (CDK9—cyclin-dependent kinase 9), responsible for TRAIL resistance of cancer cells. The study demonstrated that SNS032 and TRAIL have a synergistic effect in the treatment of cancer and can both inhibit RNA synthesis and induce apoptosis of cancer cells [9].

The second taxane family member, DTX, was also investigated in HNSCC therapy [13]. DTX resistance of TSCC was modified by EVs loaded with miR-200c. EVs miR-200c decrease chemoresistance for DTX in TSCC. A detailed mechanism for this modification is described in the section below.

Another anticancer drug, Dox, was loaded in milk-EVs to reduce its normal tissue toxicity [29]. Dox cardiotoxicity is a major complication for clinical treatment, thus the researchers engineered special DDS for therapy, i.e., Exo@Dox–EPT1, described above [29]. The EVs derived enhanced the Dox accumulation in tumour tissues and prolonged retention time. The therapeutic result of the free Dox group and EV@Dox–EPT1 were compared with the 808 nm laser irradiation group and it was noted that EV@Dox–EPT1 caused significantly more cytotoxicity in cancer cells under 808 nm laser irradiation than the free Dox group and produced synergistic effects with photochemistry.

Moreover, some authors [28] have revealed that BMEVs showed a synergistic therapeutic effect of 5-FU against OSCC in both in vitro and in vivo studies.

#### 3.6.3. RNA-Based Gene Therapy Delivered by EVs in the HNSCC Treatment

Although more than 30% of HNSCC patients demonstrate a minimal response to anticancer drugs such as cisplatin, the drug is still the first-line chemotherapeutic used for HNSCC patients. Evidence suggests miR dysregulation as one of the mediators of chemoresistance. Therefore, some research has focused on RNA-based gene therapy in HNSCC treatment. Thus, studies have revealed the role of exosomal miR and siRNA in chemotherapeutic resistance.

##### Extracellular Vesicle Derived miRs

MiRs are short (20–24 nucleotides in length) non-coding RNAs that are involved in the post-transcriptional regulation of gene expression in multicellular organisms by affecting both the stability and translation of mRNAs. In particular, miRs are essential regulators in various cellular processes, including proliferation, apoptosis, autophagy and migration [86,87]. In the reviewed articles, different types of miR loaded into EVs were assessed in HNSCC therapy.

1. MiR-9

MiR-9 was reported as the miR most related to HPV. Some studies noted that HPV + HNSCC cell-derived EVs are rich in miR-9-5p and suggested its positive role in the radiosensitivity of HNSCC cells [88,89]. MiR-9-5p polarizes macrophages into the M1 phenotype producing iNOS, which enhances tumour radiosensitivity [88]. MiR-9 significantly inhibits PPARδ (peroxisome proliferator-activated receptors subtype δ) expression in macrophages, enhancing the sensitization of HNSCC cells to radiation [22].

In summary, the exosomal miR-9 overexpression promotes HNSCC radiosensitivity, and HPV + HNSCC-derived exosomal miR-9 could be a potential treatment strategy for HNSCC.

2 MiR-18a and BART10-5p

Currently, about 15–20% of human cancers are associated with or induced by oncogenic viral factors, which drive oncogenesis mainly through impact on angiogenesis. Virus-associated tumours can be driven by herpes viruses (Kaposi’s sarcoma), HPVs (cervical cancer), EBV (nasopharyngeal carcinoma, Burkitt lymphoma, gastric carcinoma), and others. Consequently, investigators have performed research on the relationships between viruses and host miRs.

Some studies used therapy regulation by viral and host miRs to inhibit angiogenesis. Wang [24] performed a study on miR-18a and BART10-5p, one of the miRs of EBV [90]. Both these miRs promote cancer angiogenesis by downregulation of Spry3 expression. Spry3 may have a tumour-suppressor function in NPC [90]. Although two miRs targeted the same gene, they bound different sites of Spry3 and upregulated the protein expression levels of Ras, c-Raf, MEK1/2, mTOR, eIF4E1, Erk1/2, HIF1-a, mmp2, and VEGF. To inhibit NPC angiogenesis, the authors proposed antagomiR-BART10-5p and antagomiR-18a enclosed in iRGD-tagged EVs. They demonstrated that EVs simultaneously containing antagomiR-BART10-5p and antagomiR-18a suppressed the angiogenesis and growth of NPC with higher efficiency than a single treatment. These findings establish a synergistic role between virus and host miRs in the regulation of angiogenesis of virus-associated cancer.

3 MiR-34a

MiR-34a regulates the cell cycle and apoptosis. It has been demonstrated that miR-34a expression levels are low in OSCC tissues and four human OSCC cell lines (HN4, HN6, SCC-9 and CAL27) compared to normal oral tissues [91]. Therefore, the researchers focused on miR-34a delivery into cancer cells [14]. In this study, they observed that miR-34a-EVs led to a significant inhibition of cancer cell proliferation, migration and invasion by downregulation of SATB2 expression (the special AT-rich sequence-binding protein 2). SATB2 modulated the expression of those genes which regulate pluripotency and self-renewal. Overexpression of the SATB2 gene in normal epithelial cells induced transformation, suggesting its role as an oncogene [92]. SATB2 expression in OSCC was associated with metastasis and tumour progression [91].

The study suggests that miR-34a-EVs can be a therapeutic option in HNSCC via targeted inhibition of SATB2.

4 MiR-100-5p and miR-1246

Other investigated miRs were miR-100-5p and miR-1246, which play a role in HNSCC angiogenesis [93,94]. Both in vitro and in vivo studies [18] confirmed that EVs rich in these miRs inhibit cell proliferation and migration but also induce apoptosis by downregulation of several angiogenesis-related factors (such as VEGFA, MMP-9 and ANGPT) and inhibit micro-vascular formation in OSCC tumours.

5 MiR-138

MiR-138 is known to be a tumour suppressor that has been downregulated in OSCCs [95,96,97] and many other types of HNSCC [98]. The miR-138 target genes play essential roles in cell migration (e.g., ZEB2, HIF-1a), EMT (e.g., TWIST2, EZH2), cell-cycle regulation (e.g., CCND1, CCND3, FOSL1), DNA damage and repair (e.g., H2AX, XRCC1), and senescence (e.g., Sirt1, TERT) [98]. Thus, miR-138 can inhibit proliferation and invasion, induce apoptosis, and enhance the chemosensitivity in many types of cancer.

Some authors have noticed that miR-138 delivered by γδ TDEVs significantly decreased OSCC cell proliferation and viability, and induced more apoptosis, but also had an immunostimulatory effect on T cells [17]. Therefore, miR-138-EVs are beneficial in OSCC therapy.

6 MiR-144/451a

As described above, in some reviewed studies EVs were loaded with miR-144/451a in complex with chitosan [16] to construct CAs -miR-144/451a. This biomimetic system improved the miR-144/451a cluster effects of miRs on OSCC cells. Their inhibitory function was associated with miR-451a activity which may inhibit the expression of MIF (macrophage migration inhibitory factor) and CAB39 (a key regulator of aseptic 20 kinase). Both are extensively involved in tumorigenesis, promote tumour cell proliferation and differentiation, and inhibit apoptosis [99].

7 MiR-155

MiR-155 is another miR known to be a tumour-promoting factor in many cancers by regulating the BCL6/cyclin D2 axis [100]. This miR leads to the downregulation of FOXO3a (the Forkhead transcription factor O subfamily), which normally acts as a tumour suppressor gene, increases OSCC chemoresistance for DDP, and is directly associated with aggressive progression and poor prognosis in cancers [101,102].

Thus, the researchers hypothesised that the miR-155 inhibitor would be useful in OSCC therapy and noticed that it modulates the level of drug transporter ABCB1 (a drug efflux transporter protein), which is typically involved in chemoresistance in OSCC [21,103]. They observed a significantly lower ABCB1 expression in the xenograft model of OSCC treated with the miR-155 inhibitor, as compared to controls. They also demonstrated downregulation of stemness markers, such as sox2, oct4, nanog, and bmi1, in the group treated with EVs loaded with miR-155 inhibitor, suggesting a possible correlation between stemness phenotype and resistance to DDP in oral cancer.

In conclusion, the downregulation of miR-155 may potentially reverse HNSCC chemoresistance to DDP.

8 MiR-200c

Cui et al. [13] investigated EVs loaded with miR-200c for TSCC therapy [9]. MiR-200c participates in many biological processes, such as the cell cycle [104], steroidogenesis [105], vasculo-genesis [106] and EMT [107], but also sensitizes cancer cells to DTX through the PTEN/Akt pathway [108]. The authors confirmed that EVs could effectively deliver miR-200c to chemo-resistant cells and increase the sensitivity of TSCC cells to DTX [9]. DTX inhibits the development of cancer by affecting microtubules, stops the transition from metaphase to anaphase, and promotes apoptosis [46,49,109,110,111,112,113,114].

In their revised article, Cui et al. [13] observed that HNSCC cells (human tongue SCC line: HSC-3DR) showed downregulation of miR-200c, which led to decreased apoptosis in tumour cells, and higher migration and invasion associated with EMT and DNA damage. This observation was in line with miR-200c’s previously confirmed supportive role in anticancer mechanisms. They concluded that miR-200c decrease is essential for chemoresistance. MiR-200c regulated DTX resistance by targeting TUBB3 (microtubule protein βIII-tubulin) and PPP2R1B (subunit of protein phosphatase 2). Direct transfection of HNSCC cell lines using the miR-200c-encoding lentiviral vector (LV-200c) led to forced overexpression of miR-200c and resulted in higher sensitivity to DTX. miR’s role as a chemosensitizer for DTX was also confirmed in vivo by intra-tumour injection of miR-200c- EVs, which significantly suppressed tumour growth in response to DTX treatment.

To summarize, miR-200c derived by EVs decreases HNSCC resistance for DTX.

In conclusion, so far miRs are the most common molecules loaded in EVs for HNSCC therapy. The included studies confirmed miRs’ (miR-9, miR–18a, BART10-5p, miR–34a, miR-100-5p, miR-1246, miR-138, miR-144/451a) inhibitory role in HNSCC proliferation, migration and invasion, but increased apoptosis induction. Additionally, some miRs, such as miR-155, miR-200c, and miR-138, improved HNSCC chemosensitivity, and miR-9 increased the radiosensitivity of carcinoma cells. A few miRs, such as miR-144 and miR-451a, additionally had an immunostimulatory effect on T cells.

##### Extracellular Vesicles-Derived Small Interfering RNA

Small interfering RNA (siRNA), sometimes known as short interfering RNA or silencing RNA, is a molecule similar to miR which is able to regulate the expression of genes by a phenomenon known as RNAi (RNA interference). It inhibits the expression of specific genes with complementary nucleotide sequences by degrading mRNA after transcription, preventing translation.

SiRNA-based technology is a promising strategy, but difficult to apply in clinical practice, because of siRNAs’ polyanionic charge, poor stability against serum nuclease degradation, low permeability, immune response induction and toxicity. These traits can be neutralized by placing siRNAs in EVs, which deliver them to targeted cancer cells. This option was assessed by Wang [23], who examined EVs loaded with TRPP2 siRNA complex for the treatment of HNSCC. TRPP2 is a nonselective cation channel markedly increased in HNSCC [54]. TRPP2 enhances metastasis in these cells by regulating EMT. TRPP2 siRNA delivered by EVs to HNSCC cells suppressed TRPP2 protein expression levels, and as a consequence reduced EMT. TRPP2 knockdown significantly decreased ATP-induced Ca2+ release and vimentin and N-cadherin expression levels. Both N-cadherin and vimentin are overproduced in numerous cancers and recognized as prognostic biomarkers in cancero-genesis [115].

A similar strategy was used by others [15] to engineer EVs (octEVs) transferring siRNA to OSCC tumours. Specially prepared EVs along with expression of EBV proteins were loaded with siLCP1–siRNA silencing of LCP1 (lymphocyte cytoplasmic protein 1). LCP1 was recently found to be a regulator of OSCC progression and positively correlates with the primary tumoral size and regional lymph node metastasis [116]. By loading siLCP1 in octEVs, the authors achieved an OSCC tumour-suppressive effect in vitro and in vivo.

Concluding, EVs can protect and deliver siRNA into cancer cells and apply them in clinical practice. SiRNA, by regulation of the gene expression, impacts prognostic biomarkers and inhibits migration and invasion of HNSCC cells.

## 4. Discussion

In recent years, knowledge of the role of EVs in many inflammatory diseases and cancers has grown rapidly [117,118]. EVs isolated from the plasma of HNSCC patients provide information on tumour status, genetic complexity and immune dysfunction [119], but also on the molecular mechanisms involved in cancer metastasis and resistance to therapy [120,121,122].

In this article, we have discussed the potential of using membrane vesicle-based drug delivery systems (DDS) in HNSCC therapy. These novel DDS have shown promising advancements in oncology by selectively pointing to cancers, leading to better results and reduced side effects, as well as impacting tumor radio- and chemoresistance [123,124]. EVs offer several advantages, including the ability to protect, stabilize and deliver drugs precisely to the target site. Despite facing biological barriers during transport to tissues, EVs have the inherent ability to overcome this, due to their natural formation [9].

EV administration generates better tolerated and more efficient medicines. The association between the EV’s source and its tumour-targeting abilities and biodistribution opens up new ways to devise targeted therapies in order to deliver anti-tumor drugs [69].

EVs may serve as an alternative to cell-based therapeutic approaches. Compared with cell-based therapy, EVs may have a shorter lifetime in circulation. Moreover, EV treatment is associated with controllable cytokine release syndrome, and therefore minimal adverse events, good penetration within the solid tumor, and easy modification [125].

Furthermore, studies confirmed that EVs have direct anti-tumor effects on SCC and stimulate anti-tumour immunity. EVs DDS effectively reverses the multidrug resistance of tumours and improves HNSCC sensitivity for treatment. In many cases, the protection of the loaded molecules by EVs against nucleases and other enzymes in the bloodstream is critical for their use in therapies.

However, despite the growing research in this area, many issues remain incompletely clarified. These include issues necessary for safe administration, such as EVs’ pharmacokinetics, biodistribution and proper delivery of effective drug concentrations. EVs have some disadvantages also, relating to their rapid elimination from circulation, high toxicity and lack of targeting specificity, which can be managed by EV modification.

Therapeutic applications of EVs as DDS are limited due to the lack of an ideal EV donor, methods for scalable EV isolation and production, and inefficient drug loading. Moreover, cells produce different types of EVs under different conditions. Due to the heterogeneity of exosomes, recent reports suggest that there is a need to separate exosomes into distinct subpopulations [126]. Thus. scientists are looking for new technologies for the association of a specific marker with an exosome subtype and the exosome subtype with a particular function. Some authors have proposed modern microfluidic and nanotechnological advancements towards isolation and characterization of exosome subpopulations, such as phage display technology, which does not rely on the a priori definition of exosome-related markers, but is based on phage ability in exosome detection [127]. This seems to be a promising tool for the identification and isolation of disease-related exosomes, which is crucial for the effective adoption of exosomes in clinical practice.

Additionally, the development of efficient, sensitive and biocompatible EV labelling and imaging techniques is highly desired. Importantly, although EVs can be nearly non-immunogenic when used autologously, concerns remain about the immunogenicity and toxicity of unnatural DDS.

In HNSCC, the diagnosis of the disease is very often made at a late stage, with the highest mortality [128,129]. Rapid advances in the technology of using EVs as drug carriers make it hopeful that these solutions will be used in the future. The first results are impressive, but there is still a need for further research to integrate such an innovative technique into clinical practice.

## Figures and Tables

**Table 1:** Search strategy for the databases used.

Database	Number of Results	Search Strategy
Scopus	428	TITLE-ABS-KEY ((extracellular vesicle *) OR (multivesicular bodies) OR (exosome *)) AND ((squamous cell carcinoma *) AND ((head and neck) OR (larynx) OR (laryngeal) OR (nasal cavity) OR (oral) OR (pharyngeal) OR (hypopharyngeal) OR (nasopharyngeal) OR (oropharyngeal) OR (mouth))) AND ((delivery system *) AND (drug *)) Limited to: Article
Web of Science	6	AB = (((extracellular vesicle *) OR (multivesicular bodies) OR (exosome *)) AND ((squamous cell carcinoma *) AND ((head and neck) OR (larynx) OR (laryngeal) OR (nasal cavity) OR (oral) OR (pharyngeal) OR (hypopharyngeal) OR (nasopharyngeal) OR (oropharyngeal) OR (mouth))) AND ((delivery system *) AND (drug *)))
Pubmed	2	((“Extracellular Vesicles”[Mesh]) OR (“Multivesicular Bodies”[Mesh]) OR (“Exosomes”[Mesh)) AND (“Squamous Cell Carcinoma of Head and Neck”[Mesh]) AND (“Drug Delivery Systems”[Mesh])
Cochrane	0	#1 MeSH descriptor: [Extracellular Vesicles] explode all trees #2 MeSH descriptor: [Multivesicular Bodies] explode all trees #3 MeSH descriptor: [Exosomes] explode all trees #4 MeSH descriptor: [Drug Delivery Systems] explode all trees #5 MeSH descriptor: [Squamous Cell Carcinoma of Head and Neck] explode all trees #6 (#1 OR #2 OR #3) AND #4 AND #5

**Table 2:** Main data from the included studies.

	Type of Study	Type of EVs	EVs Cargo	Main Results
Cohen [12]	in vitro–A431 cell line in vivo–mice model (xenograft HNSCC)	Human MSC-EVs, A431-EVs	GNPs as labeling	1. The EVs origin has a direct impact on the tumor targeting and penetration ability. 2. MSC-EVs had superior tumor accumulation when compared to A431-EVs. 3. The use of biocompatible GNP-labeled EVs, combined with CT imaging, has the potential for application in cancer therapy.
Cui [13]	in vitro–HSC-3 cell line in vivo–mice model (xenograft TSCC)	NTECs-EVs	miR-200c	1. HSC-3DR cells showed downregulation of miR-200c. 2. The level of miR-200c was lower in TSCC lines relative to NTECs. 3. MiR-200c delivered by EVs to carcinoma cells decreased DTX resistance by targeting TUBB3 and PPP2R1B both in vitro and in vivo.
Deng [14]	in vitro–HN6 cells	HEK293T c-EVs	miR-34a	MiR-34a-EVs led to significant inhibition of HN6 cell proliferation, migration, and invasion by down-regulating SATB2 expression.
Kase [15]	in vitro–cell lines (OSCC-derived) in vivo–mice model (xenograft OSCC)	Oct-EVs	siLCP1	The silencing of LCP1 by siRNA-suppressed OSCC tumor.
Li [17]	in vitro–CAL27 in vivo–mice model (xenograft OSCC)	γδ Tcell-EVs	miR-138	1. MiR-138—γδ Tcell-EVs increased expansion and cytotoxicity of γδ Tcells resulting in inhibition of OSCC both in vitro and in vivo. 2. MiR-138—γδ Tcell-EVs were more effective than liposome transfected miR-138 and scramble-cargo γδ Tcell- EVs.
Li [16]	in vitro–UM-SCC083A, UPCI-SCC029B	M-EVs	CA-miR-144/451a	M-EVs/CA-miR-144/451a biomimetic system effectively reduced the migration, invasion, and viability of OSCC cells and was more effective than free miR-144/451a.
Liu [18]	in vitro–HUVEC in vivo–mice model (xenograft OSCC)	SHED-EVs	miR-100-5p miR-1246	1. SHED-EVs inhibited cell proliferation and migration and induced apoptosis in HUVECs. 2. SHED-EVs downregulated several angiogenesis-related factors, (VEGFA, MMP-9, and ANGPT) and inhibited micro-vascular formation in OSCC tumor.
Liu [19]	in vitro–SCC25	MSCT-EVs	SNS032	SNS032/TRAIL delivered by gelatin biomimetic system can effectively induce apoptosis of tumor cells, reduces the dosage of free drugs, and shows a high inhibitory effect on OSCC.
Qiu [20]	in vitro–SCC25 in vivo–mice model (xenograft OSCC)	MSCT-EVs	CTX/TRAIL	1. The antitumor effect of MSCT-EVs/CTX was confirmed in vitro and in vivo. 2. MSCT-EVs/CTX inhibited cell proliferation and migration and induced apoptosis in SCC cell lines and mice models.
Sayyed [21]	ex vivo–3D tumor spheroids model of UPCI-SCC-131 in vivo–mice model (xenograft OSCC)	UPCI-SCC-131-EVs	miR-155 inhibitor	1. Cisplatin-resistant OSCC tumors showed a more malignant phenotype and elevated miR-155 level. 2. Treatment with miR-155 inhibitor-loaded EVs caused chemosensitization toward cisplatin via upregulation of FOXO3a and reducing EMT.
Tong [22]	human HNSCC cell lines: SCC90, SCC47, SCC104, SAS, CAL33 HPV- cell line CAL27	HPV + HNSCC-EVs	miR-9-5p	1. HPV + HNSCC-EVs were rich in miR-9-5p. 2. HPV + HNSCC-EVs miR-9 increased the radiosensitivity of HNSCC cells by polarizing macrophages into the M1 phenotypes which produce iNOS.
Wang [23]	in vitro–FaDu cells	HEK293T cells-EVs	TRPP2 siRNA	EV/TRPP2 siRNA complexes reduced EMT in FaDu cells and inhibited FaDu cell migration and invasion.
Wang [24]	in vitro–EBV-negative and positive NPC cell lines in vivo–NPC mice model	HUVEC–EVs	antagomiR-BART10-5p; antagomiR-18a	1. IRGD-EVs-antagomiRs that contained both antagomiR-BART10-5p and antagomiR-18a attenuated the angiogenesis and growth of NPC with greater efficiency than a single treatment. 2. The findings established a synergistic role for virus and host miRs in the regulation of virus-associated tumor angiogenesis.
Wang [25]	in vitro–EBV-negative and positive NPC cell lines, radioresistant CSCs in vivo–NPC mice model	γδ TD-EVs	-	1. γδ-T-EVs can effectively interact with and kill both EBV-positive and negative NPC cells. 2. The combination therapy of radiotherapy and γδ-T-EVs was more efficient because γδ-T-EVs improved radiosensitivity by eradicating the NPC CSCs.
Yakavets [26]	ex vivo–3D tumor spheroids model of PSCC (FaDu cells)	HUVEC–EVs	mTHPC	MTHPC—EVs most effectively improved the drug delivery to PSCC due to extremely high loading capacity.
Yamayoshi [27]	in vitro–CAL27 in vivo–hind model	CAL27-EVs	ExomiR-Tracker	ExomiR-Tracker successfully inhibited the function of miR-21 and the Cal27cells growth but also inhibited tumorigenesis in vivo.
Yang [28]	in vitro – CAL 27, WSU-HN6 ex vivo–3D spheroid-model of OSCC in vivo–mice model	BMEVs	24 miRs identified in BMEVs	1. BMEVs had a synergistic therapeutic effect of 5-FU against OSCC both in vitro and in vivo. 2. BMEVs significantly downregulated NLRP3 expression. 3. BMEVs suppressed OSCC proliferation and induced apoptosis by the generation of reactive oxygen species (ROS).
Zhang [29]	in vitro–HSC-3, SCC-9, CAL-27), HCM cell line in vivo–mice model	Milk- EVs	EV@Dox loaded with EPT1 and Ce6	1. EV@Dox–EPT1 caused significantly more cytotoxicity in cancer cells under 808 nm laser irradiation than free Dox. 2. NPs produced synergistic effects of photochemistry triggered by acid TME and NIR.

BMEVs—bitter melon-derived EVs, CAL 27—an oral adeno-squamous carcinoma cell line, CAs—miR-144/451a (chitosan NPs complex with miR), Ce6—chlorin e6, CSCs—cancer stem-like cells, CTX—cabazitaxel, Dox—doxorubicin, DTX—docetaxel, EMT—epithelial to mesenchymal transition, EPT1—anthracene endoperoxide derivative, ExomiR-Tracker—anti-miR oligonucleotides+ anty CD63 antibody binds onto the surface of EVs, FaDu cells—a cell line originating from human PSCC, GNPs—gold NPs, G-SNS032—SNS032 loaded gelatin NPs, HCM—a human cardiac muscle cell line, HN6 cells—OSCC cell line, HSC-3—human TSCC cell line, MSCT-EVs—transfected MSC-EVs, MSCT-EVs/CTX—MSC–derived EVs containing TRAIL and CTX combination, mTHPC—temoporfin meta-tetra (hydroxyphenyl) chlorin, NPC—nasopharyngeal carcinoma, OSCC—oral SCC, PSCC—pharyngeal SCC, SATB2—the special AT-rich sequence-binding protein 2, SCC—squamous cell carcinoma, siLCP1—small interfering RNA of lymphocyte cytoplasmic protein 1, siRNA—small interfering RNA, TRAIL—tumour necrosis factor-related apoptosis-inducing ligand, TRPP2—transient receptor potential polycystic 2, TSCC—tongue SCC, UPCI-SCC-131—human OSCC cell lines.

## Data Availability

Not applicable.

## Supplementary Materials

The following supporting information can be downloaded at: https://www.mdpi.com/article/10.3390/pharmaceutics15051327/s1, Table S1: The risk of bias included in a systematic review assessed using the OHAT risk of bias.; Table S2: Main data from the included studies.
