# Supplementary material for: Extracellular Vesicle-Based Drug Delivery Systems for Head and Neck Squamous Cell Carcinoma: A Systematic Review
Source: Pharmaceutics. 2023 Apr 24;15(5):1327. doi: 10.3390/pharmaceutics15051327 (PMC10223025; doi:10.3390/pharmaceutics15051327)
Supplement: Supplementary file 1 [file pharmaceutics-15-01327-s001.zip › pharmaceutics-2262294-supplementary.pdf]

Table S1. The risk of bias included in a systematic review assessed using the OHAT risk of bias.

	Was administered dose or exposure level adequately randomized?	Was allocation to study groups adequately concealed?	Did selection of study participants result in appropriate comparison groups?	Were experimental conditions identical across study groups?	Were the research personnel and human subjects blinded to the study group during the study?	Were outcome data complete without attrition or exclusion from analysis?	Can we be confident in the exposure characterization?	Can we be confident in the outcome assessment?	Were all measured outcomes reported?	Were there no other potential threats to internal validity?
Cohen [12]	++	++	++	++	+	++	++	++	++	++
Cui [13]	+	-	++	++	++	+	++	++	++	++
Deng [14]	+	-	++	++	+	++	++	++	++	++
Kase [15]	+	-	++	++	+	+	++	++	++	++
Li [17]	+	-	++	++	+	++	++	++	++	++
Li [16]	+	++	++	++	+	++	++	++	++	++
Liu [18]	++	++	++	++	++	++	++	++	++	++
Liu [19]	+	++	++	++	+	++	++	++	++	++
Qiu [20]	++	++	++	++	++	++	++	++	++	++
Sayyed [21]	+	-	++	++	+	++	++	++	++	++
Tong [22]	+	-	++	++	+	++	++	++	++	++

Wang [23]	+	-	+	++	+	++	++	++	++	++
Wang [24]	+	-	+	++	+	++	++	++	++	++
Wang [25]	+	-	+	++	+	++	++	++	++	++
Yakavets [26]	+	-	++	++	+	++	++	++	++	++
Yamayoshi [27]	+	-	++	++	+	++	++	++	++	++
Yang [28]	+	-	++	++	+	++	++	++	++	++
Zhang [29]	+	-	++	++	+	++	++	++	++	++

++ indicates low, + indicates probably low, - indicates probably high, -- indicates high risk of bias

Table S2. Main data from the included studies.

	**Study group**	**Control group**	**EVs Purification**	**EVs Characterization**	**EVs Localization/ Labeling and Tracking**	**EVs Target**
Cohen [12]	A431 tumor-bearing mice injected with GNP-loaded EVs	A431 tumor-bearing mice injected with free GNPs - not encapsulated within EVs	UCG	SEM, NTA, WB for CD63	GNPs, IHC for CD63, CT, light microscopy	HNSCC in mice model
Cui [13]	HSC-3DR<br>TSCC line	HSC<br>NTECs	UCG	TEM, NTA, WB for positive (CD9 and CD63), and negative (GM130) markers	PKH67, fluorescence assays	HSC-3DR cells TSCC in mice model, genes: TUBB3 and PPP2R1B
Deng [14]	miR-34a-EVs		UCG	TEM, NTA, WB for positive (CD9 and TSG101), and negative (calnexin) markers	PKH67, Cy3	OSCC (HN6 cells), SATB2
Kase [15]	siLCP1-loaded EBI3 EVs	siControl-loaded EBI3 EVs	UCG	TEM, NTA, WB for positive markers (CD9, CD63, and CD81)	GFP, fluorescence assays, LSM, IVIS imaging	OSCC in mice model
Li [17]	miR-138 γδ Tcell-EVs applied to mice model and CAL27	both: liposome – transfected miR-138 and scramble-cargo γδ Tcell-EVs applied to mice model and CAL27	UCG	SEM, FCM, WB for positive (CD63) and negative (calnexin) markers	PKH26, fluorescence assays	OSCC
Li [16]	SCC cell lines exposed to M-EVs/CA-miR-144/451a	SCC cell lines exposed to free miR-144/451a	UCG	TEM, WB for positive markers (CD63, CD81, TSG101)	transwell assay	OSCC, MIF and CAB39 genes
Liu [18]	the SHED-EVs treated HUVEC and mice group	no treated HUVEC and mice group	EVs purification kit	TEM, NTA, FCM, WB for positive markers (CD63, TSG101)	PHK67, fluorescence assays	OSCC in mice model
Liu [19]	the combination:<br>MSCT-EV /G-SNS032	the single group:<br>G-SNS032, MSCT-EV	UCG	TEM, WB for positive markers (CD9, CD63, and CD81)		
Qiu [20]	SCC25 cell line and OSCC model injected with MSC-EVs	SCC25 cell line and OSCC model injected with MSCT-EVs/CTX	UCG	TEM, WB for positive markers (CD9, CD63)	DiR	SCC25 cell line, OSCC

Sayyed [21]	ex vivo and in vivo OSCC tumor injected with EV loaded with miR-155 inhibitor	ex vivo and in vivo OSCC tumors injected with EVs loaded with NC	UCG	SEM, NTA, WB for positive markers (CD9)	GNPs, PHK67, fluorescence assays	OSCC, FOXO3a
Tong [22]	HPV+ HNSCC cell	HPV- HNSCC cell	UCG	TEM, NTA, WB for positive (CD9, CD63, and TSG101) and negative (calnexin) markers	PHK67, fluorescence assays	PPARδ gen in macrophages
Wang [23]	EV/TRPP2 siRNA complex	siRNA only	UCG	TEM, WB for positive markers (CD9, CD63, and CD81)	PKH26, fluorescence assays	FaDu cells
Wang [24]	NPC model treated with iRGD-EVs-antagomiRs compared with EVs-antagomiRs	NPC model treated with iRGD-EVs antagomiR-BART10-5p or antagomiR-18a	UCG	DLS, TEM, LSM, WB for positive markers (CD9, CD63, and CD81)	DiD, DiI, fluorescence assays	Spry3 gen in NPC
Wang [25]	EBV positive and negative NPC model injected with γδ-T-EVs	EBV positive and negative NPC model injected PBS (control)	UCG	TEM, FCM, WB for positive markers (CD9, CD63, and CD81)	DiR, CFSE, fluorescence assays	NPC
Yakavets [26]	mTHPC - EVs in PSCC 3D model	Foslip® and mTHPC-DCL in PSCC 3D model	UCG	TEM, WB for positive markers (CD9 and CD63)	PKH67, fluorescence assays	PSCC
Yamayoshi [27]	ExomiR-Trackers against miR-21	Ctrl ExomiR-Trackers (without miR-21)	UCG	LSM, WB for positive markers (CD9, CD63 CD81, TSG101)	Alexa647, TAMRA	miR-21
Yang [28]	the BMEVs+5-FU group	the 5-FU group	UCG	WB, TEM, NTA for BMEVs positive markers: HSP70; S-adenosyl-homocysteinase; glyceraldehyde 3 phosphate dehydrogenase	DiL	OSCC
Zhang [29]	groups treated with:<br>- EV@Dox–EPT1<br>- free EVs<br>- free Dox	controls treated with PBS	UCG	DLS, TEM, WB for positive markers (CD9, CD63, TSG101)	Dox, DiL	OSCC

A431 – the A431 human epidermal carcinoma cell line, BMEVs – bitter melon-derived EVs, CFSE – carboxyfluorescein succinimidyl ester, DiD – 1,10 -dioctadecyl-3,3,30,30 -tetramethylindodicarbocyanine perchlorate, DiL – 1,1′-dioctadecyl-3,3,3′,3′-tetramethylindocarbocyanine perchlorate, DiR – 1,1′-dioctadecyl-3,3,3′,3′-tetramethylindotricarbocyanine iodide, DLS – dynamic light scattering, Dox – doxorubicin, Exo@Dox–EPT1 – exosome–doxorubicin–anthracene endoperoxide derivative NPs, FaDu cells – a cell line originating from human PSCC, FCM – flow cytometry, Foslip® – mTHPC in conventional liposomes, GFP – the green fluorescent protein, GNPs – gold nanoparticles, HEK293T cells – human embryonic kidney cells, HNSCC – head and neck SCC, HSC – hematopoietic stem cell, HSC-3DR – docetaxel-resistant HSC-3 line, HSP70 – heat shock protein 70, HUVEC – human umbilical vein endothelial cell, IHC – immunohistochemical staining, LSM - a confocal laser scanning microscope, mTHPC – temoporfin meta-tetra (hydroxyphenyl) chlorin, mTHPC-DCL – mTHPC drug-in-cyclodextrin-in-liposomes, M-EVs – macrophage-derived exosomes, MSC – mesenchymal stem cells, MSCT-EVs – transfected MSC-EVs, NC – normal control, NLRP3 – NOD-like receptor family pyrin domain containing 3, NPC – nasopharyngeal carcinoma, NTA –

nanoparticle tracking analysis, octEVs – engineered OSCC-targeted EVs that express a transmembrane EBV protein (Induced-3 [EBI3]), OSCC – oral SCC, PBS – phosphate-buffered saline, PKH 26/67 – red fluorescence linker, PSCC – pharyngeal SCC, ROS – reactive oxygen species, SATB2 – the special AT-rich sequence-binding protein 2, SCC – squamous cell carcinoma, SEM – scanning electron microscope, SHED – stem cells of human deciduous exfoliated teeth, TAMRA – Carboxytetramethylrhodamine, TECs – normal tongue epithelial cells, TSCC – tongue SCC, UCG – ultracentrifugation, UPCI-SCC-131 – human OSCC cell lines, WB – Western blot
